# An Emerging Screening Method for Interrogating Human Brain Function: Tutorial

**DOI:** 10.2196/37269

**Published:** 2023-04-27

**Authors:** Gustavo Sudre, Anto I Bagić, James T Becker, John P Ford

**Affiliations:** 1 Brain FIT Imaging, LLC Unadilla, NY United States; 2 University of Pittsburgh Comprehensive Epilepsy Center, Department of Neurology Pittsburgh, PA United States; 3 Departments of Psychiatry, Neurology, and Psychology, University of Pittsburgh Pittsburgh, PA United States

**Keywords:** screening, brain function, cognition, magnetoencephalography, MEG, neuroimaging, tutorial, tool, cognitive test, neuroimaging, signal, cognitive function

## Abstract

Cognitive decline can be observed due to a myriad of causes. Clinicians would benefit from a noninvasive quantitative tool to screen and monitor brain function based on direct measures of neural features. In this study, we used neuroimaging data from magnetoencephalography (with a whole-head Elekta Neuromag 306 sensor system) to derive a set of features that strongly correlate with brain function. We propose that simple signal characteristics related to peak variability, timing, and abundance can be used by clinicians as a screening tool to investigate cognitive function in at-risk individuals. Using a minimalistic set of features, we were able to perfectly distinguish between participants with normative and nonnormative brain function, and we were also able to successfully predict participants’ Mini-Mental Test score (*r*=0.99; *P*<.001; mean absolute error=0.413). This set of features can be easily visualized in an analog fashion, providing clinicians with several graded measurements (in comparison to a single binary diagnostic tool) that can be used for screening and monitoring cognitive decline.

## Introduction

The expanding cohort of older people with deteriorating cognitive functions poses a burden to the affected individual, the individual’s family, and society [1]. Standardized neuropsychiatric tests are helpful, but getting a view of the actual brain function of each individual would be better. For example, do all individuals with a similar cognitive test score have the same cognitive deficits? Does brain function remain stable in all individuals over a very short time horizon? Is the response to a stimulus the same for each repetition of the stimulus? The answers to these questions may be important to monitor therapeutic interventions as diverse as drug treatment and counseling.

To date, several techniques have been used to interrogate human brain function. They range from neurocognitive tests [2] to neuroimaging techniques [3-5] and viral vectors [6], among others. Although neurocognitive tests have been the golden standard for clinical diagnosis, they do not measure brain function directly, and therefore, there has been a need for more applicable monitoring tools.

Among neuroimaging techniques, functional magnetic resonance imaging is a noninvasive technique that measures changes in blood flow to different areas of the brain [7]. This can be used to create a map of brain activity and identify which areas of the brain are active during different tasks. Functional magnetic resonance imaging offers superior spatial resolution, but it is dependent on the time scale of blood deoxygenation, and therefore, cannot offer the millisecond resolution seen in magnetoencephalography (MEG). Conversely, electroencephalography (EEG) measures the electrical activity of the brain using electrodes placed on the scalp [8]. This can be used to identify abnormal patterns of brain activity and diagnose conditions such as epilepsy. Although it does offer temporal resolution comparable to MEG, the electrical currents are damped by the skull, making it harder to assess the location of the sources of brain signals. Finally, positron emission tomography is a brain imaging technique that uses radioactive tracers to measure brain activity [9]. This can be used to identify changes in brain metabolism or blood flow, which can be indicative of different conditions. Positron emission tomography can be used to interrogate a myriad of metabolic disorders, but one of its downsides is the invasive nature of the procedure, which is not the case for the previously mentioned imaging techniques.

Viral vectors have recently emerged as a powerful tool for investigating the neural circuits underlying human cognition and possibly developing new treatments for neurological and psychiatric disorders [10]. By introducing genetic material into specific populations of neurons, viral vectors can be used to manipulate neural activity and study the functional connectivity between different brain regions. Different types of viral vectors, such as lentiviruses and adeno-associated viruses, offer different advantages and limitations for gene delivery. Recent studies have used viral vectors to manipulate the activity of specific brain regions involved in cognitive processes, such as decision-making and memory formation [11,12]. However, the use of viral vectors in human studies presents several ethical and practical challenges that must be carefully considered [13].

In this work, we recognize the vast clinical experience with electrocardiogram (ECG) in the analysis of human heart function, which shows that the examination of timing and fidelity of individual depolarizations is useful in establishing normative versus nonnormative heart electrical function [14]. We applied similar metrics and their correlates to brain function in a cohort of older people with normative and nonnormative heart electrical function using MEG, a clinically established neurophysiologic technique [15] that offers superior time and spatial resolution to comparable modalities.

## Methods

### Recruitment and Scanning Protocol

A total of 10 participants had cognitively normal brain function, and 10 age-matched participants had nonnormal cognitive function (mean age 75.1, SD 6.4 years). All participants were right-handed and tested for hearing preservation as a requirement for participation. Participants underwent 2 MEG scans (using whole-head Elekta Neuromag 306 sensor system) approximately 45 minutes apart (run 1 and run 2) as well as a battery of neuropsychological assessments. In the scanner, participants were presented with a random series of standard and deviant tones (50-ms tone duration, every 2.5 seconds; 5:1 proportion) for a total of 250 tones.

### Ethical Considerations

The protocol was approved by the Institutional Review Board of the University of Pittsburgh. The study data were deidentified prior to the analysis.

### Signal Processing and Channel Selection

For each participant, the data in all 306 MEG sensors were band-pass filtered between 1 Hz and 30 Hz to keep most of the variance in the power of the recordings and also to remove any slow drifts in the data, normally related to recording artifacts. The timing of the presentation of each standard tone was determined, and data beginning 100 milliseconds prior to presentation to 500 milliseconds after the presentation represented 1 epoch. All signal processing analysis was conducted using the MNE-Python package (version 0.23.0) and scikit-learn (version 0.24.1).

We analyzed data from the ipsilateral sensor that showed the most stable response to the tones across epochs, as the ipsilateral response to simple sound stimuli has been shown to display significant delays in different peaks of the neural response [16]. Within a predetermined pool of 12 gradiometers that commonly capture auditory responses, we selected the sensor with the least variability across epochs. Specifically, variability was calculated by computing the evoked response (ie, response averaged over epochs) after randomly splitting all epochs into 2 halves and calculating the correlation between the 2 evoked responses. We repeated this process thousands of times and defined the most stable sensor to be the one with the highest median correlation between the 2 evoked responses.

### Feature Estimation

**Figure 1:**
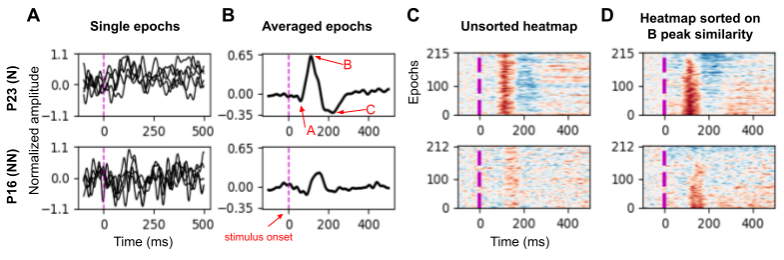
Data for representative participants with normative and nonnormative brain functions (P23 and P16, respectively). (A) the first 5 epochs for that run. (B) the average of all epochs. In the heatmap (C and D), individual epochs are placed on the vertical axis, and color indicates the normalized signal amplitude (red for positive and blue for negative).

With a single channel selected, the first step was to determine the timing of each of the 3 peaks (Figure 1). Peak time windows are defined by their onset and offset and are calculated using the averaged response (ie, an individual has peak onset and offset times for a single run, not single epochs). We defined peak latency as the time point in which the peak reaches its maximum absolute value. As noticed in Figure 1A, inferring peak properties in a single epoch basis is challenging due to signal variability. Although timing features can be estimated using the average over epochs (Figure 1B), much information is lost. The heatmap (Figure 1C and Figure 1D) offers an alternative way to visualize and analyze the data. For example, when epochs are sorted on B peak similarity, the percentage of epochs with B peaks and the variability among epochs can be a distinguishing feature between participants with normative and nonnormative brain function.

Using the averaged response, the method starts by finding the B peak latency, which is the maximum absolute value of the signal within the 110- to 190-millisecond window. From that point, it goes back in time (toward 0 ms, when the stimulus was presented), until it finds the time point in which the signal first reaches a value lower than twice the baseline standard deviation (where baseline is the signal 100 ms before the stimulus presentation). The algorithm then repeats the same procedure going forward in time from B peak latency, again looking for the time point in which the signal first returned to twice the baseline standard deviation.

The onset of the A peak was fixed at 50 milliseconds due to the instability of the signal relative to the peak magnitude, and the A peak offset was defined the same as the B peak onset. Similarly, C peak onset was chosen to be the same as the B peak offset. Finally, the C peak offset was chosen as the time point in which the signal recovered to the same value seen at the B peak offset plus 2 baseline standard deviations or to the most positive value if the signal never gets back to that amplitude.

**Figure 2:**
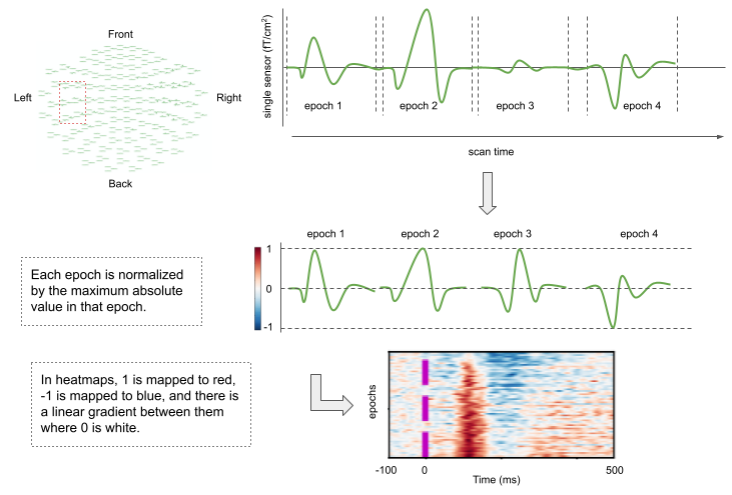
Schematic from single sensor signal to heatmap. A single sensor out of a pool of 12 ipsilateral gradiometers is selected based on signal stability. Epochs are amplitude normalized prior to display in heatmap. Purple marks in heatmap mark time 0, when stimulus is presented.

With the onset and offset of each of the 3 peaks determined (Figure 2), we then established what percentage of the epochs displayed the given peak using heatmaps. A heatmap is organized such that the epochs (vertical axes) are arranged on the basis of a specific feature. For example, Figure 1D shows a heatmap where individual epochs are sorted based not on data acquisition sequence but on signal similarity within the B peak time window for that individual. The epoch with the strongest signal within that time window is at the bottom of the heat map. The epoch with the most similar signal within the time window to that bottom epoch is just above it and so on. Signal similarity between epochs can be calculated in a variety of ways. Here we used a nonlinear spectral embedding to reduce the signal of one epoch (t time points representing a t-dimensional space) into a single dimension. Specifically for spectral embedding, we constructed a k-nearest neighbors graph with n nodes (for n observations) and projected it to a single dimension by calculating a spectral decomposition of the graph Laplacian. Nodes that are connected in the original graph are clustered together. The procedure effectively summarizes the n by t data into n values, which can then be further sorted based on their Euclidean distance in that single-dimensional space to organize the heatmap.

**Figure 3:**
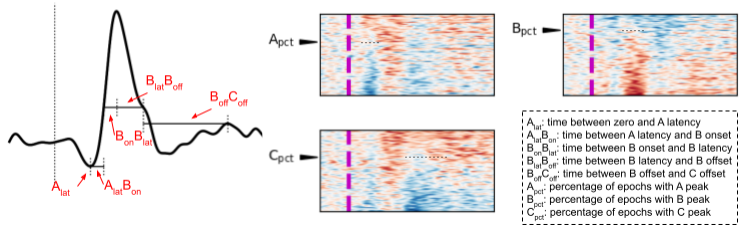
Features placed on a diagram of an idealized averaged response and heatmaps. Latency corresponds to the time point in which the peak reaches it maximum absolute value. B peak onset is the time in which the B peak surpasses 2 standard deviations of the baseline signal (time <0), and the offset is when the signal returns to a value below that same threshold. C peak offset is the time when the signal returns to the amplitude at B peak offset or when it reaches its most positive value (if that return does not happen). Variability features measure the degree of dissimilarity across epochs within a time window.

Once organized based on peak similarity, we counted the epochs with that given peak used in sorting (Figure 3). We first sorted the heatmap based on the peak window. That actual heatmap was then spatially correlated with every possible ideal heatmap, representing possible heatmaps where 0%-100% of the epochs have the B peak. Each ideal heatmap has a linear gradient within the peak window, where the bottom epoch has a value of 1 and the last epoch is 0. The ideal heatmap with the highest correlation to the actual heatmap estimates the percentage of epochs with the peak. In other words, we looked for the ideal heatmap most similar to the actual heatmap within that time window. We then repeated the same procedure for the remaining 2 peaks.

Finally, a similar procedure to how channel stability is calculated was also used to calculate B peak variability, except that the correlation between the two averaged responses was computed using only the signal between the B onset and offset for the individual. In addition, variability was defined as 1 minus the median correlation to better illustrate the concept (ie, higher variance in the signal corresponding to higher feature value).

### Feature Evaluation

To evaluate this set of features, we used both a classification and a prediction approach. First, we used a Gaussian Naive Bayes classifier to define the most concise, but yet intuitive, set of features that could differentiate between participants with normative and nonnormative brain function. We conducted a progressive evaluation of prediction models with an increasing number of features. Specifically, we started with all possible models with 2 features and calculated how well a Gaussian Naive Bayes model could predict a left-out subject (leave-one-out cross validation). We further increased the model requirements to 3 all the way to the 13 possible features. Then, we used a Partial Least Squares model (version 2.7-3; R package PLS) to predict the Mini-Mental Test scores using the set of 13 features. The absolute value of each feature was used in this prediction model.

## Results

**Figure 4:**
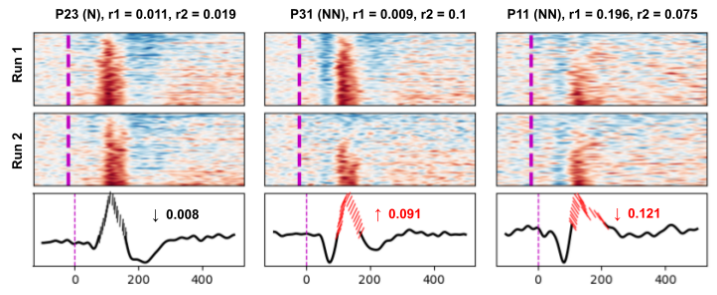
Run-to-run differences in B peak variability. Although the variability remains similar across both runs for P23, it increases for P31 and decreases for P11. The slash angle over the B peak in averaged response (bottom row) indicates the amount and direction of B peak variability difference between runs. A_pct_: A peak; B_pct_: B peak; C_pct_: C peak; N: normative; NN: nonnormative; r1: run 1; r2: run 2.

We report finding a simple MEG-derived metric that accurately distinguishes between the subject groups. We found 3 distinct peaks (ie, A, B, and C) in the averaged neural response to the tone (Figures 1-3). By using the heatmaps for visualization (Figure 4), the percentage of epochs with B peaks and the variability among epochs in the B-peak time window can be distinguishing features between participants with normative and nonnormative brain function. Specifically, while the variability remains similar across both runs for P23, it increases for P31 and decreases for P11. Forward-leaning slashes for P31 indicate a significant increase in variability between runs, and backward-leaning slashes for P11 indicate a decrease, with the red color representing values outside the normative range.

**Figure 5:**
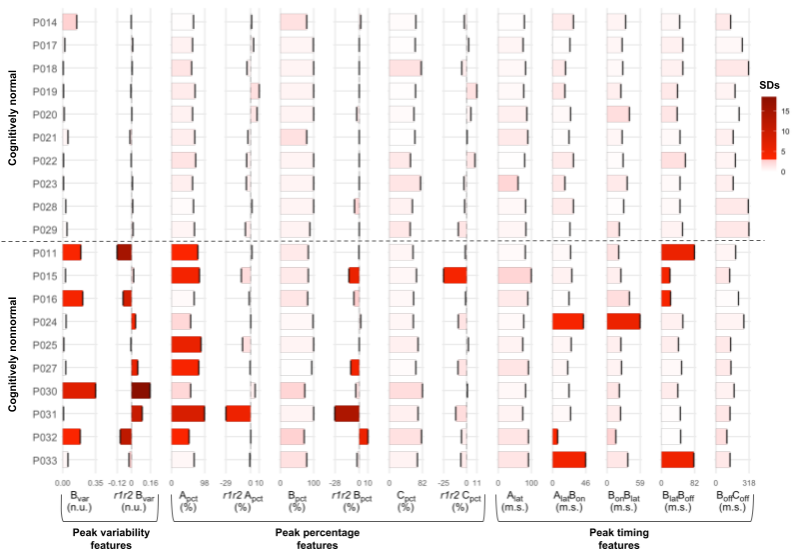
Barplots showing subjects and features. Each column shows the subject values for that feature, and the bar color indicates the outlier Grubb score (number of standard deviations from normal subjects). Subjects are presented in alphabetical order within group, and features are organized based on the information they carry. A_lat_: A peak latency; A_pct_: A peak; B_off_: B offset; B_on_: B onset; B_pct_; B peak; B_var_: B peak variability; C_pct_; C peak; r1r2: run 1 run 2 (eg, r1r2A_pct_ stands for the difference in percentage of A peaks between run 1 and run 2).

**Figure 6:**
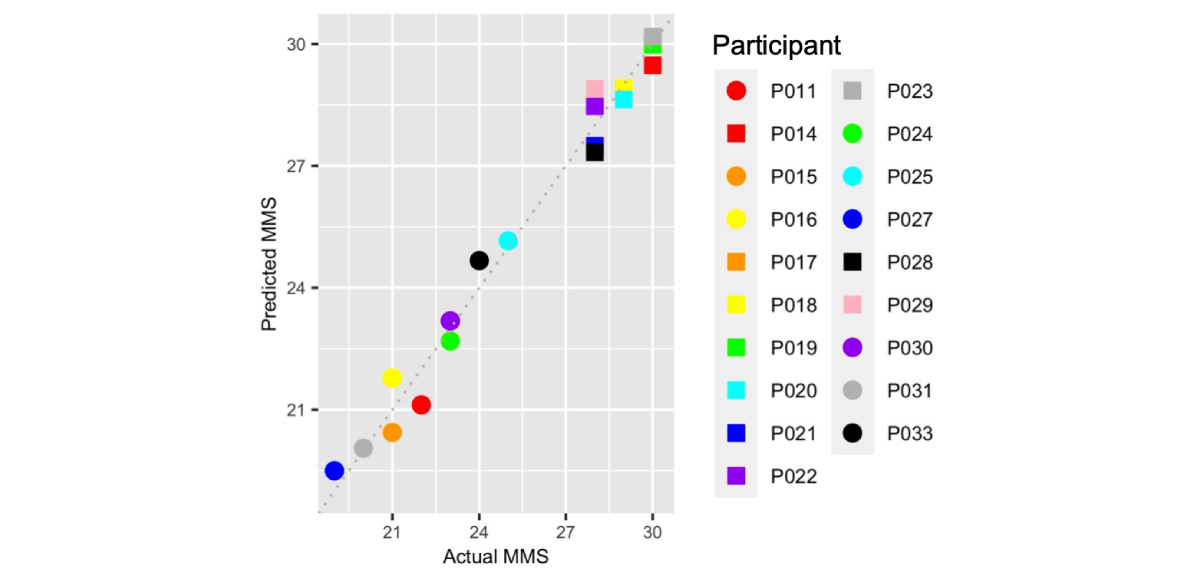
Predicting Mini-Mental Test score (MMS) using partial least squares regression (12 components, mean absolute error=0.413; *r*=0.99; *P*<.001). The absolute value of the set of 13 features was used to train the model; predicted scores are displayed against their actual MMS values for each individual subject.

Overall, 13 features describing peak variability, percentage, and timing were used to quantify the response (Figure 5). Features were computed using the average signal over epochs and also the heatmap, a visual representation of all individual epochs. Single features showed participants with nonnormative brain function as significant outliers when compared to normative participants (Figure 5) (outlier Grubb score >3 [17]). A model with as few as 4 specific features could perfectly classify between left-out participants with normative and nonnormative brain function: (1) run-to-run difference in B peak variability, (2) percentage of epochs with A peaks, (3) interval between A peak latency and B peak onset, and (4) interval between B peak latency and offset. It is important to note that these 4 critical features do not have the same weight in the model. For instance, run-to-run difference in B peak variability has twice as much weight as the second ranked feature (percentage of epochs with A peaks). Finally, using a Partial Least Squares model to reduce dimensionality, the original set of 13 features was also able to predict subjects’ Mini-Mental Test scores (Figure 6; *r*=0.99; *P*<.001; mean absolute error=0.413). Results for the model with 12 components are displayed, but even the simplest Partial Least Squares model with a single component yielded highly significant results (*r*=0.83; *P*<.001; mean absolute error=1.62).

## Discussion

This study measured the neural response to a tone, and 3 distinct peaks (A, B, and C) were identified. A total of 13 features were used to quantify the response, and a model with 4 specific features was able to perfectly classify between left-out participants with normative and nonnormative brain function. Using a Partial Least Squares model to reduce dimensionality, the original set of 13 features was also able to predict subjects’ Mini-Mental Test scores. Overall, these findings demonstrate the potential for using MEG-derived metrics to accurately identify cognitive impairments in individuals.

It has been recently proposed that complex cognitive disorders, such as Alzheimer Disease, could be conceptualized as multiple subtypes [18], reflecting a lack of common and systematic pathology. The features described in this work can help characterize such subgroups. In fact, this lack of a single overarching feature that was present in all participants with nonnormative brain function is one of the key findings in our data set.

The strongest feature in separating participants with normative and nonnormative function was the variability of B signal shape between the 2 runs. Participants with normative function showed very little change between the 2 runs. For participants with nonnormative brain function, there was a subgroup with more variability in run 2 compared to run 1, consistent with fatigue between the runs. There was a second subgroup with decreased variability in run 2, suggesting increased cognitive engagement from the rest period for these participants.

Other features of participants with nonnormative brain function might also have readily identifiable behavioral correlates, such as a loss of adaptation response to the stimulus with increased percentage of epochs with A peaks [19] and consequent neural fatigue evident in loss of run-to-run difference in percentage of epochs with A peaks and B peaks variability in these participants, as noted above. In addition, the only 2 participants in this category with a prolonged interval from A peak latency to B peak onset were among the 4 nonnormative group participants with normal percentage of epochs with A peaks, a result consistent with a possible protective effect from the delay on cognitive fatigue and representative of the compounded interactions of complex systems captured in the MEG response [20].

Although the clinical use of MEG has seen a steady increase in medical hospitals [21], especially in the localization of seizures, the authors recognize that the need for an MEG machine is a limitation of the proposed screening tool. The development of more mobile MEG devices is promising [22] and would indeed accelerate the adoption of the proposed tool by clinicians. It is also possible that similar features as the ones identified in this work would be present in the signal of more practical measuring devices, such as ECG caps, which would further expand the use of this monitoring method among the clinical community.

Similar to how ECG is used to assess heart function, the combination of features identified here provides an objective and subject-specific clinical tool for clinicians to monitor an older patient’s cognitive status. Such a screening device may prove useful in assessing response to therapies as well as cognitive function in at-risk individuals.

## Abbreviations

ECG: electrocardiogram
MEG: magnetoencephalography

## Data Availability

All data, associated protocols, and codes used can be accessed by contacting the corresponding author.
